# Triptolide Inhibits the Biological Processes of HUVECs and HepG2 Cells via the Serine Palmitoyltransferase Long Chain Base Subunit 2/Sphingosine-1-Phosphate Signaling Pathway

**DOI:** 10.1155/2022/9119423

**Published:** 2022-11-18

**Authors:** Lulu Jia, Shengnan Zhu, Mingfei Zhu, Lingyue Huang, Siyuan Xu, Yuqin Luo, Juan Xiao, Huazhen Su, Shaoyuan Huang, Qinyou Tan

**Affiliations:** ^1^Clinical Pharmacy & Pharmacology Research Institute, Affiliated Hospital of Guilin Medical University, Guilin 541001, China; ^2^Laboratory of Hepatobiliary and Pancreatic Surgery, Affiliated Hospital of Guilin Medical University, Guilin 541001, China; ^3^China-USA Lipids in Health and Disease Research Center, Guilin Medical University, Guilin 541001, China; ^4^Guangxi Key Laboratory of Molecular Medicine in Liver Injury and Repair, Affiliated Hospital of Guilin Medical University, Guilin 541001, China; ^5^Guangxi Health Commission Key Laboratory of Basic Research in Sphingolipid Metabolism Related Diseases, Affiliated Hospital of Guilin Medical University, Guilin 541001, China

## Abstract

Triptolide (TP) has demonstrated innumerous biological effects and pharmacological potential against different cancer types. Hepatocellular carcinoma has a high incidence in men, and its incidence is increasing year by year. Studies have shown that angiogenesis plays an important role in the formation of tumors and that angiogenesis is closely related to tumor growth and metastasis. Deregulation of sphingolipids signaling has been associated with several pathological conditions, including cancer. In the present study, we aimed at exploring the potential molecular mechanism of TP's antivascular and antitumor effects in vitro from the perspective of sphinolipids. Human umbilical vein endothelial cells (HUVECs) and HepG2 cells were, respectively, treated with different concentrations of TP and transfected. Then, the effect of HUVECs on HepG2 cells was investigated using a three-dimensional coculture model system. CCK-8 assay was performed for cell proliferation. Cell migration and invasion abilities were assessed using the transwell assay. Cell adhesion and tube formation were detected by Matrigel. RT-PCR and western blotting were used to detect the mRNA and protein expression. The S1P production was measured via ELISA assay. Our results showed that TP inhibited HUVECs and HepG2 cells proliferation, migration, invasion, adhesion, angiogenesis, and serine palmitoyltransferase long chain base subunit 2 (SPTLC2) expression; upregulating SPTLC2 facilitated the proliferation, migration, invasion, adhesion, angiogenesis, and sphingosine-1-phosphate (S1P) production of HUVECs and HepG2 cells, while interfering with SPTLC2 expression inhibited them; HUVECs facilitated the proliferation, migration, invasion, S1P production, S1PR1, and S1PR2 expression of HepG2 cells, while S1PR3 expression was decreased. In conclusion, SPTLC2 may be associated with the antivascular and antitumor effects of TP, and SPTLC2 is expected to become a new marker for tumor therapy. HUVECs can promote the proliferation, migration, and invasion of HepG2 cells, which may be related to the S1P/sphingosine-1-phosphate receptor (S1PR) signaling pathway.

## 1. Introduction

Hepatocellular carcinoma is a cancer that has a high incidence in men [1]. The pathogenesis of liver cancer is a very complicated process that includes a series of related processes mediated by various risk factors, such as chronic viral hepatitis, alcohol abuse, nonalcoholic steatohepatitis, and type 2 diabetes [2]. Studies show that angiogenesis plays an important role in the formation of tumors by providing nutrients to tumor cells and that angiogenesis is closely related to tumor growth and metastasis [3]. In addition, these new vascular tissues can provide blood and nutrients for the continued growth of tumors, and tumor tissues can further promote the regeneration of blood vessels in a variety of ways, thereby forming a vicious cycle. Thus, based on the principles of modern medicine, it is believed that blocking the vascular tissues of tumors can cause the tumors to “starve to death.” Therefore, finding new antivascular and antitumor targets involved in the pathogenesis of liver cancer is a key issue that needs to be addressed. Only by blocking the nutritional supply of tumor tissues and inhibiting their further growth can liver cancer be fundamentally overcome.

In recent years, further study has found that in addition to being the basic component of the cell membrane, sphingolipids also participate in a variety of signal transduction pathways and play important roles in the development of various diseases. The metabolism of sphingolipids is a key pathway in cancer biology, and their metabolites, namely, ceramide, sphingosine, and S1P, together regulate tumor cell death, proliferation, and drug resistance, as well as angiogenesis and inflammation [4]. Ceramide is produced by the hydrolysis of sphingolipids, which can participate in the *de novo* synthesis of ceramide from the precursor dihydroceramide, which is converted to ceramide by dihydroceramide desaturase to induce tumor cell apoptosis [5]. Ceramide is hydrolyzed by a ceramidase to produce sphingosine, which is phosphorylated by sphingosine kinases 1 and 2 (SK1 and SK2) to produce S1P. S1P also binds to and activates the G protein-coupled receptor family S1P receptor 1-5 (S1PR1-5), which regulates the biological activity of cells [4]. S1P/S1PR signaling pathway plays an important role in maintaining normal tissue physiological functions, such as cell growth and division [6, 7]. In addition, many studies have shown that S1P/S1PRs signaling can promote the process of tumor development, such as promoting the proliferation and migration of tumor cells, promoting angiogenesis, and the formation of tumor microenvironment [7, 8]. However, different S1PRs may have different biological effects. Studies have shown that S1PR2 plays a positive role in apoptosis and autophagy, while S1PR3 has the opposite effect [9]. Studies in many cancer cell lines indicate that S1P induces proliferation [10] and inhibits ceramide-induced apoptosis [6]. Ceramide is a key factor in sphingolipid metabolism, and serine palmitoyltransferase (SPT) is a key enzyme for the *de novo* synthesis of ceramide. In mammals, SPT is a heterodimer composed of two subunits, namely, serine palmitoyltransferase long chain base subunits 1 and 2 (SPTLC1 and SPTLC2), and serine palmitoyltransferase long chain base subunit 3 (SPTLC3) is the third subunit that was discovered in 2009 [11]. SPT plays an important role in the regulation of growth [12].

TP is one of the main active ingredients extracted from the roots, stems, and leaves of *Tripterygium wilfordii*. TP is a small molecule compound with antitumor, antiangiogenic, anti-inflammatory, and proapoptotic properties [13]. It is found that TP exerts strong inhibitory effect on the biological processes of liver [14, 15], ovarian [16–19], lung [20–22], gastric [23, 24], and breast [25, 26] cancer cells.

Based on the facts mentioned above, this study explored the effects of TP on liver cancer from the three perspectives of antivascular effects, tumor suppression, and the tumor microenvironment and identified a connection between the antiliver cancer effects of TP and sphingolipids. This experiment first studied the effect of TP on HUVECs and its possible mechanism, explored the effect of TP on angiogenesis, and investigated the effect of TP on new angiogenesis targets. Second, this experiment studied the effect of TP on HepG2 cells and further explored a new target for TP to exert its effects against liver cancer. As members of the tumor microenvironment, vascular endothelial cells not only form vascular nutrition tumor tissue but also penetrate the entire tumor tissue. Therefore, do endothelial cells themselves exert a certain effect on tumor cells? Then, this study further explored the interaction between HUVECs and HepG2 cells in a three-dimensional coculture model and its possible mechanism and studied the effect of TP on the coculture system.

## 2. Materials and Methods

### 2.1. Cell Culture

HUVECs were purchased from the North Branch of the Institute of Biotechnology, and HepG2 cells were purchased from the Chinese Academy of Sciences. The cells were cultured in Dulbecco's modified Eagle's medium (DMEM) supplemented with 10% fetal bovine serum (FBS) and 1% (*v*/*v*) penicillin-streptomycin and were maintained at 37°C in a humidified 5% CO_2_ incubator.

### 2.2. Cell Proliferation Assay

Cell viability was determined by CCK-8 assay. In monoculture systems, HUVECs and HepG2 cells were adjusted to a density of 4 × 10^4^ cells/mL and plated into 96-well plates (100 *μ*L/well). HUVECs were treated with TP (0, 12.5, 25, or 50 nM), DMSO (negative control), and endostatin (positive control, 8 mg/L) for 24, 48, and 72 h. HepG2 cells were treated with TP (0, 1, 2, or 4 *μ*M) and DMSO (negative control) for 24 and 48 h. In the monoculture transfection system, HUVECs and HepG2 cells were divided into a SPTLC2 small interfering RNA group (siR-SPTLC2), SPTLC2 plasmid group (SPTLC2), negative control group (siR-NC/SPTLC2-NC), and a blank control group (blank). In addition, HUVECs and HepG2 cells were treated with 25 nM and 2 *μ*M TP for 24 h. In the coculture systems, 2 × 10^3^ HepG2 cells (200 *μ*L/well) were added to the upper chamber of the 24-well coculture system, and 5 × 10^3^ HUVECs (500 *μ*L/well) were added to the lower chamber (coculture). DMEM (500 *μ*L/well) containing 10% FBS was added to the lower chamber in the control group (nonculture). Cells were incubated for 1 to 4 days. In the coculture dosing system, HUVECs were treated with TP (0, 12.5, 25, or 50 nM) and DMSO (negative control) for 24 h, and then, the HUVECs were washed with PBS. These treated HUVECs were cultured with HepG2 cells in the coculture system for 24 h as described.

In monoculture or monoculture transfection systems, the supernatants were removed, and 100 *μ*L of CCK-8 medium (CCK-8 reagent: DMEM = 1 : 10) was added to the wells. In the coculture and coculture dosing systems, the upper chamber was transferred to a new 24-well plate containing 500 *μ*L of CCK-8 medium (CCK-8 reagent: DMEM = 1 : 10). After 1 to 4 h, the absorbance was measured at 450 nM. We used the OD value to indicate the proliferative ability of the cells.

### 2.3. Cell Migration and Invasion Assay

The migration ability of HUVECs and HepG2 cells was determined using a 24-well two-compartment transwell assay. Cells in the monoculture, monoculture transfection, coculture, and coculture treatment systems were cultured as described for the proliferation assays. Each group of HUVECs and HepG2 cells (3 × 10^4^/200 *μ*L) was resuspended in serum-free DMEM. Two hundred microliters of the cell suspension was added to the upper chamber of the transwell, and 500 *μ*L of complete medium was added to the lower chamber. After 24 h, the upper chamber was removed and washed with phosphate buffered saline (PBS) 3 times. Then, the cells were fixed with 4% tissue cell fixative for 1 h, washed again with PBS 3 times, stained with 0.1% crystal violet for 30 min, and washed again with PBS 3 times. The cells inside the chamber were gently removed with a cotton swab, and the remaining cells were ultimately photographed with a microscope (100×). When imaging was completed, each group of chambers was destained for 5 min in 500 *μ*L of a 10% (*v*/*v*) acetic acid solution, and the absorbance was measured at 550 nM.

The cell invasion assay followed a procedure similar to the cell migration assay except that the transwell membrane was pretreated with Matrigel and the HepG2 cells density was adjusted to 3 × 10^5^/200 *μ*L.

### 2.4. Cell Adhesion Assay

The precooled Matrigel (50 *μ*L/well) was placed in a precooled 96-well plate. Then, 2% bovine serum albumin (BSA, 100 *μ*L) was added to each well and incubated for 1 h for blocking. The HUVECs in the monoculture and monoculture transfection systems were cultured as described in the proliferation assay. HUVECs (3 × 10^4^/100 *μ*L) were seeded in 96-well plates. After 1 h, the supernatants were removed, and 100 *μ*l of CCK-8 medium (CCK-8 reagent: DMEM = 1 : 10) was added to the wells. After 1 to 4 h, the absorbance was measured at 450 nM.

### 2.5. Cell Tube Formation Assay

Matrigel was plated in 96-well culture plates and allowed to polymerize at 37°C in 5% CO_2_ humidified for 30 min. HUVECs were digested with 0.25% trypsin to prepare a cell suspension, adjusted to a cell density of 8 × 10^4^/100 *μ*L, and added to a 96-well plate with Matrigel. Then, 100 *μ*L of cell suspension was added per well, and the cells were imaged after 4-8 h (100×). The quantification of tube formation was carried out by counting the number of branch points.

### 2.6. PCR Array and RT-PCR Assay

Total RNA was extracted from cells using TRIzol Universal (TIANGEN, Beijing, China) according to the manufacturer's protocol. Absorbance was measured, and the purity of RNA was assessed using values of 280/260 and 260/230. cDNA was prepared using the PrimeScript RT reagent Kit (TIANGEN, Beijing, China) according to the manufacturer's instructions. Genetic screening was performed using an RT^2^ Profiler PCR Array kit (QIAGEN, Maryland, USA). The PCR array plate included 48 genes, including 40 target genes and 8 control genes. PCR amplification was performed under the following conditions: 40 cycles at 95°C for 15 s and 60°C for 60 s. GAPDH was used as an internal control. GAPDH forward primer (5′-3′): CAGGAGGCATTGCTGATGAT; GAPDH reverse primer (5′-3′): GAAGGCTGGGGCTCATTT; SPTLC2 forward primer (5'-3'): CAGATTGCTTGAGGCCAGGAAGTTC; *SPTLC2* reverse primer (5'-3'): AGTGGTGTGATCTTGCTCATTGC.

### 2.7. Western Blotting

After HUVECs and HepG2 cells were treated with drugs or transfected following the above conditions, the protein was extracted for subsequent experiments. The cells were subsequently lysed with RIPA buffer (Solarbio, Beijing, China). Equivalent amounts of protein were separated by 10% SDS-PAGE and transferred to a PVDF membrane. The membranes were blocked in 5% nonfat milk in TBST for 1 h at room temperature and then incubated with primary antibodies at 4°C overnight. The primary antibodies used in this study were purchased from Abcam (Abcam, Cambridge, UK) and included antiserine palmitoyltransferase antibody (ab23696), anti-S1P1 antibody (ab233386), anti-S1PR2 antibody (ab220173), and anti-S1P3 antibody (ab126622). The secondary antibodies were incubated at room temperature. The membrane incubates secondary antibodies at room temperature. After the secondary antibody was incubated, the membrane was observed with ECL plus and X-ray film. Finally, the protein concentration was analyzed using ImageJ.

### 2.8. Enzyme-Linked Immunosorbent Assay (ELISA)

In the monoculture transfection system, HUVECs and HepG2 cells were cultured as described in the proliferation assay. 8 × 10^5^ HUVECs (2 mL/well) and 1 × 10^6^ HepG2 (2 mL/well) cells were seeded in 6-well plates. After the cells were cultured for 24 h, the supernatants were collected. In the coculture system, 2 × 10^5^ HepG2 cells (2 mL/well) were added to the upper chamber of the 6-well coculture chamber, and 1 × 10^5^ HUVECs (1 mL/well) were added to the lower chamber. After the cells were cocultured for 1 to 4 days, the supernatants were collected. S1P levels were detected by a sphingosine-1-phosphate ELISA kit (Echelon, Salt Lake, USA) according to the manufacturer's protocol.

### 2.9. Statistical Analysis

All the data are presented as the mean ± standard deviation (SD). GraphPad Prism 5.0 software was applied for statistical analysis, and the significance between groups was ascertained by one-way ANOVA compared with the least significant difference. When the *P* value was less than 0.05, the analysis was considered statistically significant.

## 3. Results

### 3.1. TP Inhibited HUVECs Proliferation, Migration, Adhesion, and Angiogenesis

**Figure 1:**
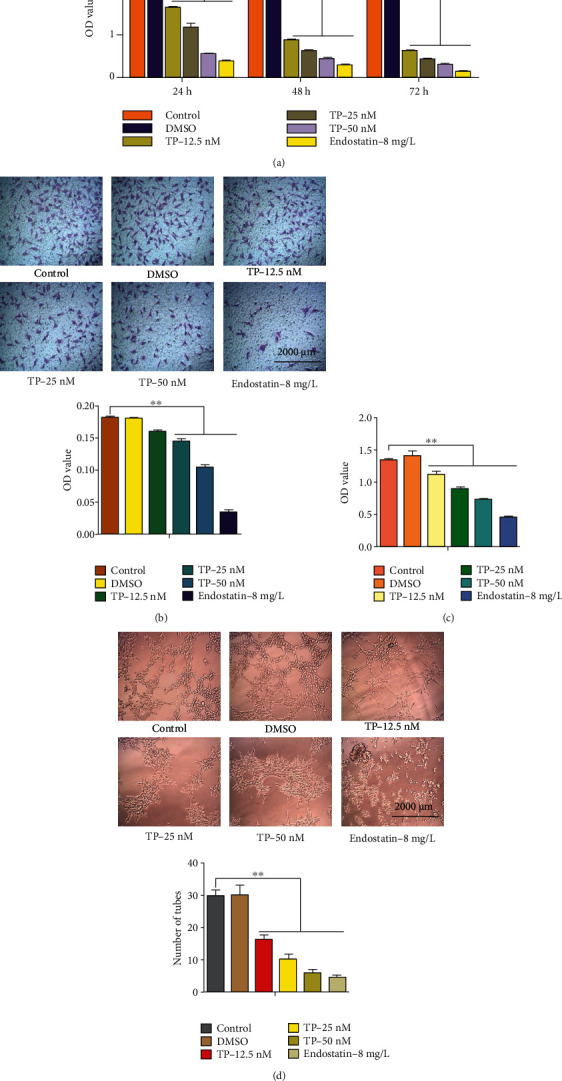
TP inhibited the proliferation, migration, adhesion, and angiogenesis of HUVECs. (a) A CCK-8 assay was used to determine HUVECs proliferation after treatment with TP (0, 12.5, 25, or 50 nM), DMSO, and endostatin (8 mg/L) for 24, 48, and 72 h. After treating HUVECs with TP (0, 12.5, 25, or 50 nM), DMSO, and endostatin (8 mg/L) for 24 h, the migration (b), adhesion (c), and angiogenesis (d) of HUVECs were measured. ^∗^*P* < 0.05, ^∗∗^*P* < 0.01 versus the control group.

First, the effects of TP on HUVECs proliferation were assessed. The results (Figure 1(a)) showed that the TP inhibited the proliferation of HUVECs in a dose- and time-dependent manner, endostatin (positive control) significantly inhibited the proliferation of HUVECs, and DMSO (negative control) had no significant effect on the proliferation of HUVECs. After treating HUVECs with TP (0, 12.5, 25, or 50 nM), DMSO, and endostatin (8 mg/L) for 24 h, the migration (Figure 1(b)), adhesion (Figure 1(c)), and angiogenesis (Figure 1(d)) of HUVECs were measured. The migration capacity of HUVECs was measured in a 24-well transwell chamber. The migrated cells in each chamber were destained (500 *μ*L and 10% acetic acid), and the absorbance was measured at 550 nM to indicate the number of migrated cells. The data showed that TP inhibited HUVECs migration, adhesion, and angiogenesis in a dose-dependent manner, endostatin (positive control) significantly inhibited HUVECs migration, adhesion, and angiogenesis, and DMSO (negative control) had no significant effect on HUVECs migration, adhesion, or angiogenesis.

### 3.2. TP Downregulated the Expression of SPTLC2 in HUVECs

**Figure 2:**
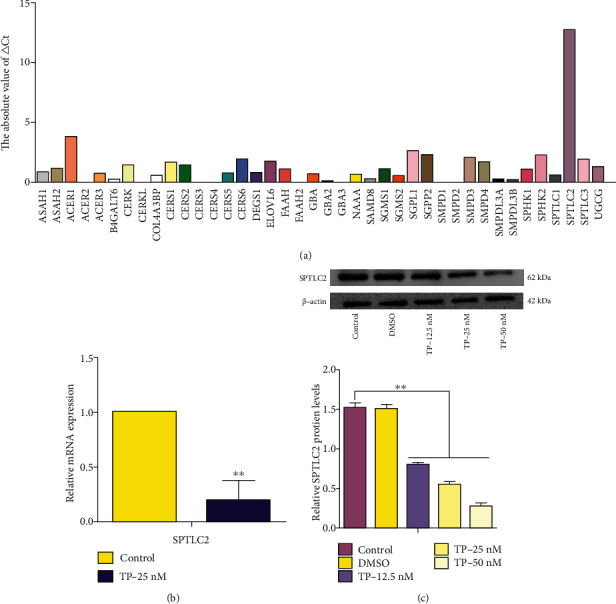
TP significantly downregulated the expression of SPTLC2 in HUVECs. (a) HUVECs were treated with TP (25 nM) for 24 h, and the changes in related genes were screened by PCR array assay. The results are shown as the absolute value of ΔCt. (b) HUVECs were treated with TP (25 nM) for 24 h, and the expression of SPTLC2 mRNA was detected by RT-PCR assay. (c) HUVECs were treated with TP (0, 12.5, 25, or 50 nM) and DMSO (negative control) for 24 h, and the protein expression of SPTLC2 in HUVECs was detected by western blotting assay. ^∗^*P* < 0.05, ^∗∗^*P* < 0.01 versus the control group.

The results (Figure 2(a)) showed that TP (25 nM) had a regulatory effect on a variety of sphingolipid genes, but among all the genes, SPTLC2 exhibited the largest changes in expression, and SPTLC2 was likely to be a new target of TP. The results are shown as the absolute value of ΔCt. Therefore, we chose SPTLC2 for subsequent studies. HUVECs were treated with TP (25 nM) for 24 h, and the mRNA expression of SPTLC2 in HUVECs was measured by RT-PCR assay. The data (Figure 2(b)) revealed that TP could significantly inhibit the expression of SPTLC2 in HUVECs. HUVECs were treated with TP (0, 12.5, 25, or 50 nM) and DMSO (negative control) for 24 h, and the protein expression of SPTLC2 was detected by western blotting assay. The data (Figure 2(c)) showed that TP could inhibit the expression of SPTLC2 in HUVECs in a dose-dependent manner, and DMSO had no significant effect on SPTLC2.

### 3.3. SPTLC2 Affected the Proliferation, Migration, Adhesion, Angiogenesis, and S1P Production of HUVECs

**Figure 3:**
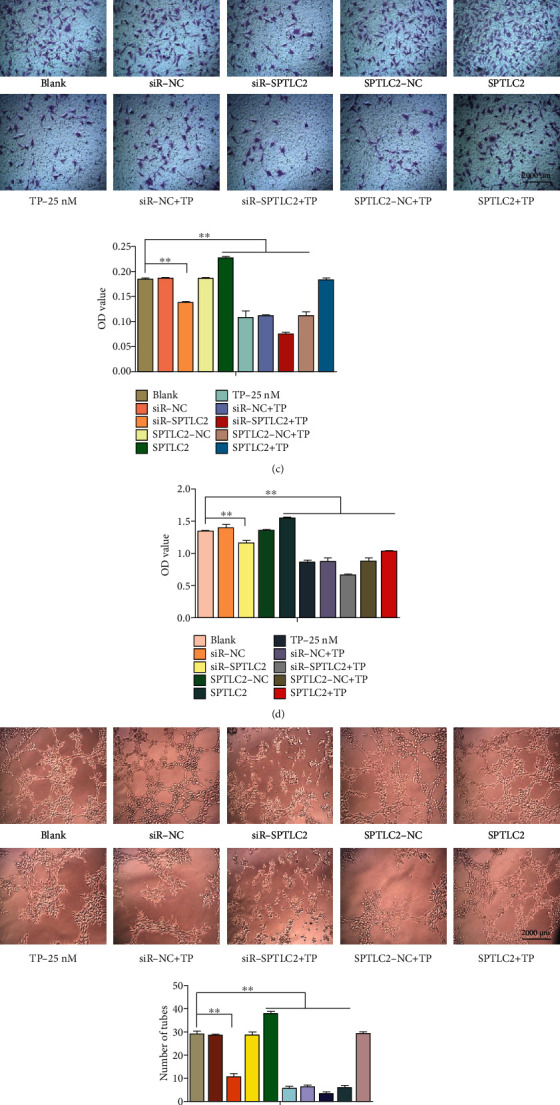
SPTLC2 can affect the proliferation, migration, adhesion, angiogenesis, and S1P production of HUVECs. After HUVECs were transfected with siR-NC (SPTLC2 small interfering RNA negative control), siR-SPTLC2 (SPTLC2 small interfering RNA), SPTLC2-NC (SPTLC2 plasmid negative control), or SPTLC2 (SPTLC2 plasmid), the protein expression of SPTLC2 was detected by western blotting assay (a), and the proliferation (b), migration (c), adhesion (d), angiogenesis (e), and S1P production (f) were measured. ^∗^*P* < 0.05, ^∗∗^*P* < 0.01 versus the blank group.

The results (Figure 3(a)) showed that the protein expression of SPTLC2 was significantly inhibited in the siR-SPTLC2 (SPTLC2 small interfering RNA) group, the protein expression of SPTLC2 was significantly increased in the SPTLC2 (SPTLC2 plasmid) group, and the protein expression of SPTLC2 was not significantly changed in the siR-NC (SPTLC2 small interfering RNA negative control) and SPTLC2-NC (SPTLC2 plasmid negative control) groups. After HUVECs were transfected with siR-NC, siR-SPTLC2, SPTLC2-NC, or SPTLC2, their proliferation (Figure 3(b)), migration (Figure 3(c)), adhesion (Figure 3(d)), angiogenesis (Figure 3(e)), and S1P production (Figure 3(f)) were measured. The results showed that siR-SPTLC2 could significantly inhibit the proliferation, migration, adhesion, angiogenesis, and S1P production of HUVECs; SPTLC2 could significantly increase the proliferation, migration, adhesion, angiogenesis, and S1P production of HUVECs; and siR-NC and SPTLC2-NC had no significant effect on the proliferation, migration, adhesion, angiogenesis, and S1P production of HUVECs. On this basis, the proliferation, migration, adhesion, and angiogenesis of HUVECs in each group were further inhibited after treatment with TP (25 nM). Based on these results, SPTLC2 may regulate various biological processes in HUVECs by regulating the production of S1P.

### 3.4. TP Inhibited the Proliferation, Migration, Invasion, and SPTLC2 mRNA and Protein Expression in HepG2 Cells

**Figure 4:**
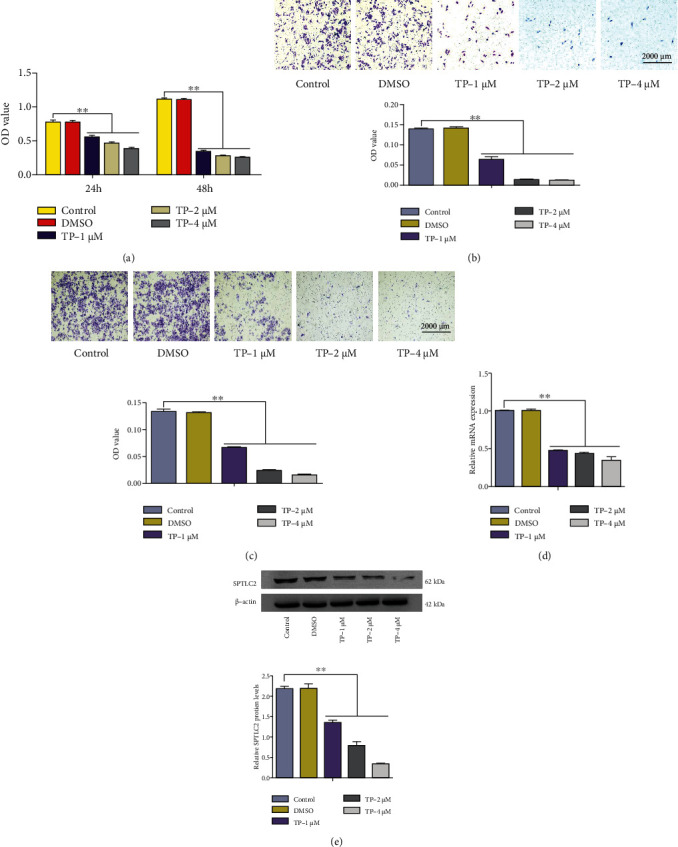
TP inhibited the proliferation, migration, invasion, and SPTLC2 mRNA and protein expression in HepG2 cells. (a) A CCK-8 assay was used to determine HepG2 cell proliferation after treatment with TP (0, 1, 2, or 4 *μ*M) and DMSO (negative control) for 24 and 48 h. After treating HepG2 cells with TP (0, 1, 2, or 4 *μ*M) and DMSO (negative control) for 24 h, the migration (b), invasion (c), and SPTLC2 mRNA (d) and protein (e) expression of HepG2 cells were measured. ^∗^*P* < 0.05, ^∗∗^*P* < 0.01 versus the control group.

Figure 4(a) presents that TP inhibited the proliferation of HepG2 cells in a dose- and time-dependent manner, and DMSO had no significant effect on the proliferation of HepG2 cells. After treating HepG2 cells with TP (0, 1, 2, or 4 *μ*M) and DMSO (negative control) for 24 h, the migration (Figure 4(b)), invasion (Figure 4(c)), and SPTLC2 mRNA (Figure 4(d)) and protein (Figure 4(e)) expression of HepG2 cells were measured. The migration or invasion capacity of HepG2 cells was assessed in a 24-well transwell chamber, the migrated or invaded cells in each chamber were destained (500 *μ*L and 10% acetic acid), and the absorbance was measured at 550 nM to indicate the number of migrated or invaded cells. The data showed that TP inhibited HepG2 cell migration, invasion, and SPTLC2 mRNA and protein expression in a dose-dependent manner, and DMSO had no significant effect on HepG2 cell migration, invasion, or SPTLC2 mRNA and protein expression.

### 3.5. SPTLC2 Affected the Proliferation, Migration, Invasion, and S1P Production of HepG2 Cells

**Figure 5:**
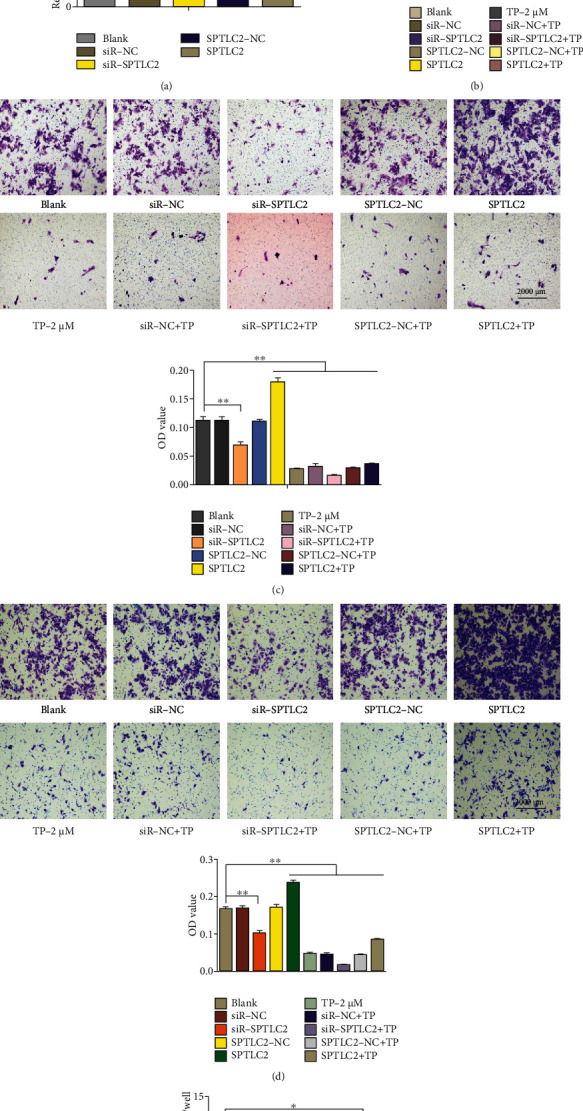
SPTLC2 can affect the proliferation, migration, invasion, and S1P production of HepG2 cells. After HepG2 cells were transfected with siR-NC (SPTLC2 small interfering RNA negative control), siR-SPTLC2 (SPTLC2 small interfering RNA), SPTLC2-NC (SPTLC2 plasmid negative control), and SPTLC2 (SPTLC2 plasmid), the protein expression of SPTLC2 was detected by western blotting assay (a), and the proliferation (b), migration (c), invasion (d), and S1P production (e) were measured. ^∗^*P* < 0.05, ^∗∗^*P* < 0.01 versus the blank group.

The results (Figure 5(a)) showed that the protein expression of SPTLC2 was significantly inhibited in the siR-SPTLC2 (SPTLC2 small interfering RNA) group, the protein expression of SPTLC2 was significantly increased in the SPTLC2 (SPTLC2 plasmid) group, and the protein expression of SPTLC2 was not significantly changed in the siR-NC (SPTLC2 small interfering RNA negative control) and SPTLC2-NC (SPTLC2 plasmid negative control) groups. After HepG2 cells were transfected with siR-NC, siR-SPTLC2, SPTLC2-NC, and SPTLC2, their proliferation (Figure 5(b)), migration (Figure 5(c)), invasion (Figure 5(d)), and S1P production (Figure 5(e)) were measured. The results showed that siR-SPTLC2 could significantly inhibit the proliferation, migration, invasion, and S1P production of HepG2 cells; SPTLC2 could significantly increase the proliferation, migration, invasion, and S1P production of HepG2 cells; and siR-NC and SPTLC2-NC had no significant effect on the proliferation, migration, invasion, and S1P production of HepG2 cells. On this basis, the proliferation, migration, and invasion of HepG2 cells were further inhibited after treatment with TP (2 *μ*M). Based on these results, SPTLC2 may regulate various biological processes of HepG2 cells by regulating the production of S1P.

### 3.6. HUVECs May Induce the Proliferation, Migration, and Invasion of HepG2 Cells via the S1P-S1PRs Pathway

**Figure 6:**
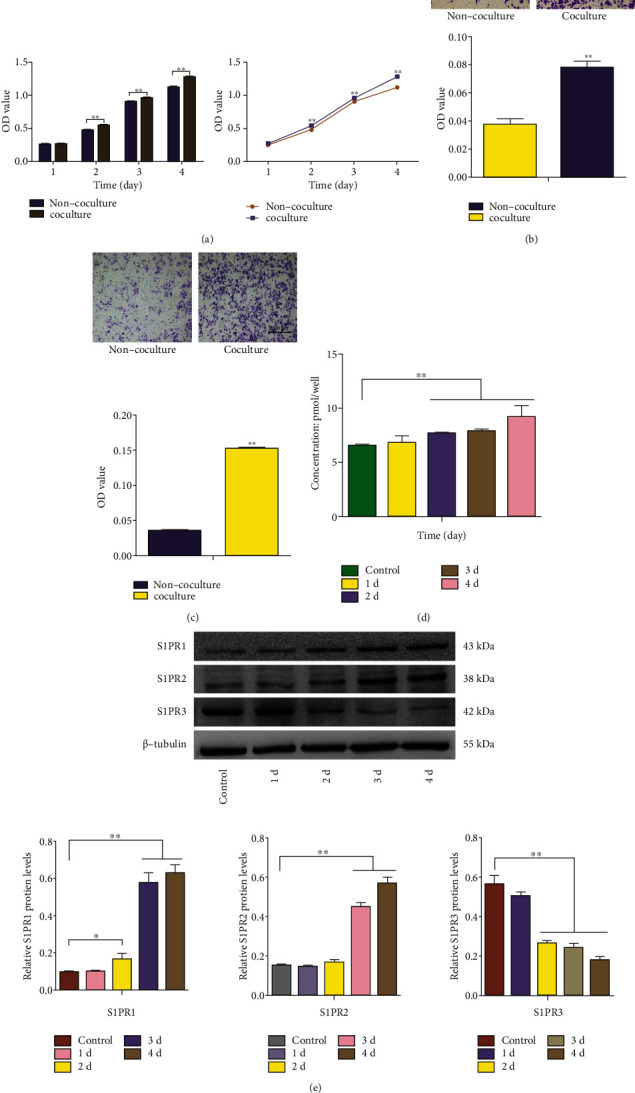
HUVECs may induce the proliferation, migration, and invasion of HepG2 cells via the S1P-S1PR pathway. (a) HUVECs and HepG2 cells were cocultured for 1 to 4 days, and a CCK-8 assay was used to determine HepG2 cells proliferation. HUVECs and HepG2 cells were cocultured for 24 h, and their migration (b) and invasion (c) were measured. HUVECs and HepG2 cells were cocultured for 1 to 4 days, and the S1P content (d) in the coculture system and the S1PR1, S1PR2, and S1PR3 protein expression (e) in HepG2 cells were measured. ^∗^*P* < 0.05, ^∗∗^*P* < 0.01 versus the noncoculture or control group.

To verify the effect of HUVECs on the proliferation, migration, and invasion of HepG2 cells, HUVECs and HepG2 cells were cocultured to further detect the proliferation, migration, and invasion of HepG2 cells. In the noncocultured or control group, HepG2 cells were added to the upper chamber of the 24-well coculture chamber, and DMEM was added to the lower chamber. In the cocultured group, HepG2 cells were added to the upper chamber of the 24-well coculture chamber, and HUVECs were added to the lower chamber. The HUVECs and HepG2 cells were cocultured for 1 to 4 days. The results (Figure 6(a)) showed that HUVECs promoted the proliferation of HepG2 cells, and their proliferative effects increased as the coculture time increased. HUVECs and HepG2 cells were cocultured for 24 h, and the data showed that HUVECs promoted the migration (Figure 6(b)) and invasion (Figure 6(c)) of HepG2 cells (100×). HUVECs and HepG2 cells were cocultured for 1 to 4 days, and the data showed that the content of S1P (Figure 6(d)) in the coculture system and the protein expression of S1PR1 and S1PR2 (Figure 6(e)) in HepG2 cells increased, while the protein expression of S1PR3 (Figure 6(e)) decreased gradually.

### 3.7. TP Inhibited the Proliferation, Migration, and Invasion of HepG2 Cells Induced by HUVECs

**Figure 7:**
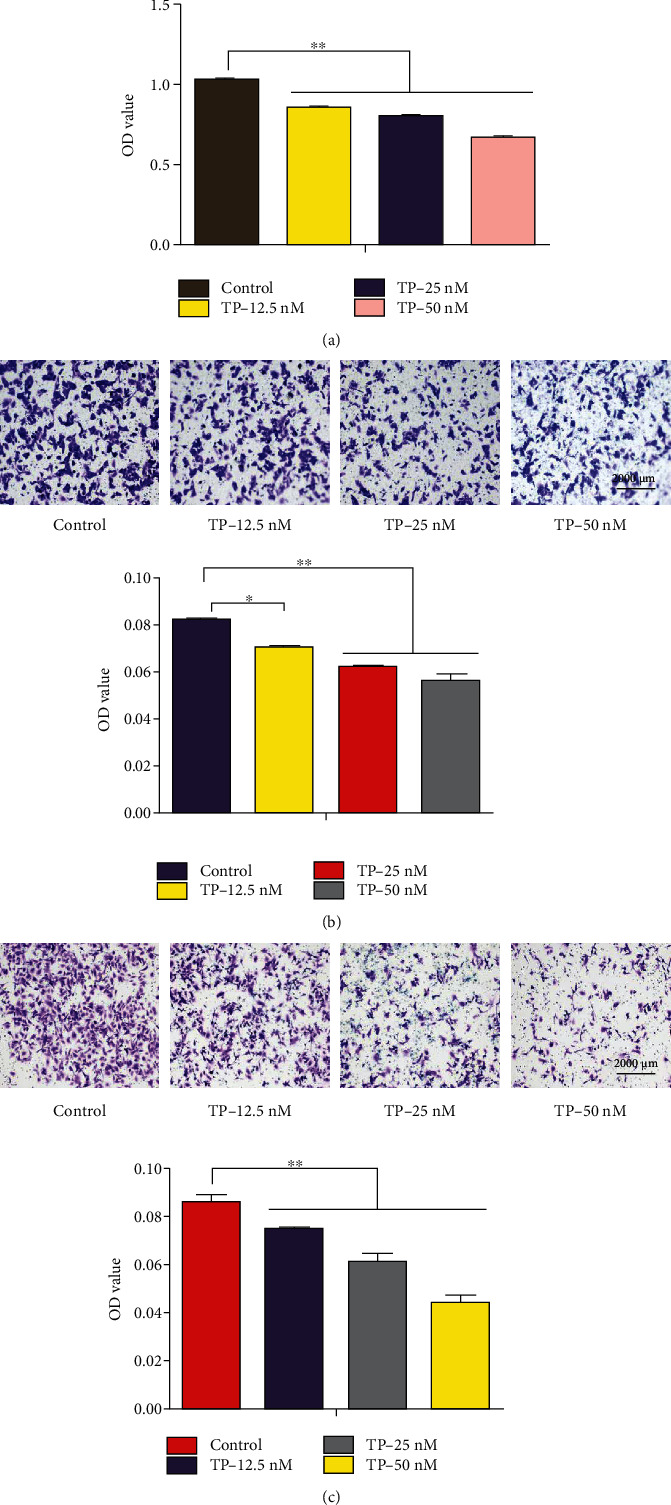
TP inhibited the proliferation, migration, and invasion of HepG2 cells induced by HUVECs. HUVECs were treated with TP (0, 12.5, 25, or 50 nM) for 24 h, the media was changed to remove the effects of the drugs, and the treated HUVECs were cocultured with HepG2 cells in a transwell coculture chamber. The data showed that when HUVECs were treated with TP, its ability to promote the proliferation (a), migration (b), and invasion (c) of HepG2 cells was significantly inhibited. ^∗^*P* < 0.05, ^∗∗^*P* < 0.01 versus the control group.

HUVECs were treated with TP (0, 12.5, 25, or 50 nM) for 24 h, the media was changed to remove the effects of the drug, and the treated HUVECs were cocultured with HepG2 cells in a transwell coculture chamber. HepG2 cells were added to the upper compartment and treated HUVECs were added to the lower compartment. The data showed that when HUVECs were treated with TP, its ability to promote the proliferation (Figure 7(a)), migration (Figure 7(b)), and invasion (Figure 7(c)) of HepG2 cells was significantly inhibited.

## 4. Discussion

**Figure 8:**
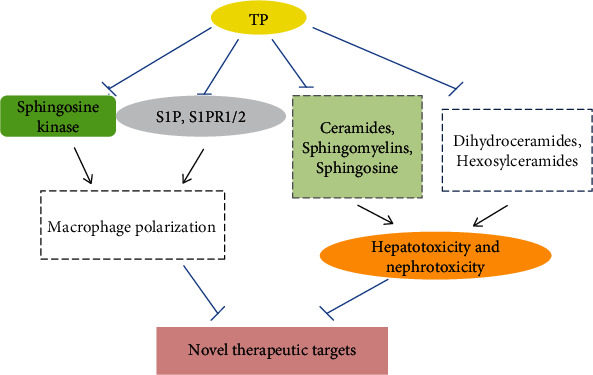
The TP effects on sphingolipid signaling pathway.

Angiogenesis is known to play an important role in tumor growth and metastasis. The newly formed vascular tissue can not only lead to the metastasis of tumor cells but also provides a continuous nutritional supply to tumor tissue [27, 28]. Therefore, according to modern medicine principles, the removal of tumor blood vessel tissue can “starve” the tumor. Therefore, in addition to inhibiting the development of tumor tissue, in the treatment of tumors, it is more important to control the formation of new blood vessels. TP is one of the most popular antitumor drugs in recent years. TP inhibits not only tumor angiogenesis but also various tumor biological processes [29–31]. In recent years, studies have found that sphingolipids are not only the basic components of cell membranes but also participate in a variety of signal transduction pathways and play important roles in the development of various diseases, especially tumors [4]. However, the antitumor effect of TP has mainly focused on the tumor suppressor gene p53 [14, 32], microRNAs [33, 34], P-glycoprotein [35], mitogen-activated protein kinases/extracellular signal-regulated kinase (MAPK/ERK) [32], and high-mobility group box 1 (HMGB1) [36], while studies on the sphingolipid signaling pathway are relatively lacking. The signal diagram which indicates the TP effects on sphingolipid signaling pathway is shown in Figure 8. Based on these facts, this research first studied the mechanism underlying the antivascular and antiliver cancer effects of TP. The experimental data showed that TP could downregulate the expression of SPTLC2 in HUVECs and HepG2 cells, and further transfection experiments showed that the biological behavior of the cells was significantly inhibited after SPTLC2 knockdown, while the biological behavior of the cells was significantly enhanced after SPTLC2 upregulation. In addition, the downregulation of SPTLC2 expression inhibited the production of S1P in two kinds of cells, while upregulation of SPTLC2 expression increased the production of S1P. Based on the above experimental results, SPTLC2 is likely to be a new antivascular and antitumor target of TP, and the regulatory effect of SPTLC2 on cells is likely to be realized by indirectly regulating the production of S1P. SPTLC2 is likely to be a new target for tumor inhibition via sphingolipid-related pathways. Perhaps, the high expression of SPTLC2 is closely related to the development of tumors, and it is very likely to become one of the markers for auxiliary tumor examination.

Angiogenesis is associated with many types of tumors, especially solid tumors, such as liver and breast cancer. As components of the tumor microenvironment, vascular endothelial cells can not only form vascular nutrient tumor tissue but can also penetrate the whole tumor tissue. Therefore, vascular endothelial cells themselves have a certain effect on promoting biological processes of tumor cells. A coculture model is a common method to study interactions between cells. Costa et al. [37] studied the correlation between human hematopoietic stem/progenitor cells and mesenchymal stem/stromal cells using a coculture model. Chen et al. [38] used liver tumors cocultured with stellate cells to study drug resistance and intercellular interactions. Bernhardt et al. [39] studied the interaction between primary human osteoclasts and mature human osteoclasts in a coculture model. Machado et al. [40] investigated the effect of HepG2 cells on endothelial cells through a coculture model. Few studies have investigated the effect of endothelial cells on tumor cells. Thus, this research designed coculture experiments with HUVECs and HepG2 cells. The data showed that the proliferation, migration, and invasion of HepG2 cells cocultured with HUVECs were significantly enhanced compared with HepG2 cells cultured alone. Mechanistic studies have shown that HUVECs may secrete S1P and then act on S1PR in HepG2 cells. Our results showed that HUVECs could promote the expression of S1PR2 in HepG2 cells, thereby promoting their proliferation, migration, and invasion. However, Ghosal et al. [9] found that S1PR2 could promote apoptosis and autophagy, which is contrary to our results. This also indicates that the effect of S1PR2 may be opposite in different cells, and its effect may be different or even opposite when the cell environment and other factors are different [41]. Previous reports have shown that S1PR3 plays a positive role in cell proliferation [42]. However, our data showed that HUVECs could inhibit the expression of S1PR3 in HepG2 cells, which may be beneficial for their proliferation, migration, and invasion. Our results suggest that S1PR3 may also have two-sidedness, and more mechanisms need to be further verified by subsequent experiments. This discovery further draws attention to the research field of the effects of vascular endothelial cells on tumor cells and enables us to determine that vascular endothelial cells can also promote the biological processes of tumor cells. These findings provide a theoretical basis for further research on the effects between vascular endothelial cells and tumor cells. However, this conclusion has only been verified in HepG2 cells, and these effects need to be further studied in other tumor cells and animal models. This conclusion may suggest a potential new target for the clinical treatment of tumors. In addition to tumor cells, vascular endothelial cells are also the focus of tumor therapy. Although TP can inhibit a variety of tumor cells through a variety of pathways, its high hepatotoxicity limits its clinical application. Zhao et al. [43] found that TP can induce apoptosis of liver cells by acting on phosphatidylinositide 3-kinases (PI3K), MAPK, tumor necrosis factor *α* (TNF-*α*), and p53 signaling pathways and further affect the metabolism of glycerophospholipids, fatty acids, leukotrienes, purines, and pyrimidines, which eventually lead to liver toxicity. Hasnat et al. [44] found that TP can cause mitochondrial dysfunction and mitochondrial autophagy by affecting the generation of reactive oxygen species (ROS), thereby causing toxicity to L02 hepatocytes. Inhibition of the liver toxicity induced by TP is a key issue that urgently needs to be resolved in order to better take advantage of its antitumor effect. However, there are few studies in this field. Tan et al. [45] found that licorice root extract and magnesium isoglycyrrhizinate can inhibit the liver toxicity of TP through the Nrf2 pathway.

In conclusion, the results of this research indicated that TP inhibited the biological processes of HUVECs and HepG2 cells by regulating the SPTLC2-S1P axis and that HUVECs could promote the biological behavior of HepG2 cells. These findings are helpful to further understand the antivascular and antitumor effects of TP via the sphingolipid pathway and to further reveal the role of sphingolipids in the development of tumors.

## 5. Conclusion

In vitro experiments showed that TP could inhibit the biological behavior of HUVECs and HepG2 cells by downregulating the expression of SPTLC2. SPTLC2 is a promising target for tumor inhibition in the future. HUVECs may promote the biological behavior of HepG2 cells through S1P/S1PR signaling pathway, and TP can inhibit these processes. These results suggest that vascular endothelial cells may affect the prognosis of tumor.

## Data Availability

The authors confirm that the data supporting the findings of this study are available within the article.
